# P-2007. Accuracy and Precision of Self-Administered Audiograms Using a Smartphone Application

**DOI:** 10.1093/ofid/ofaf695.2171

**Published:** 2026-01-11

**Authors:** Nabin K Shrestha, Tricia Bravo, Omar Mehkri, Carmen Jamis

**Affiliations:** Cleveland Clinic, Cleveland, Ohio; Cleveland Clinic, Cleveland ,OH, Cleveland, Ohio; Cleveland Clinic, Cleveland ,OH, Cleveland, Ohio; Cleveland Clinic, Cleveland, Ohio

## Abstract

**Background:**

Risk of hearing loss is a major concern for infectious disease physicians treating patients with prolonged courses of potentially ototoxic antimicrobials. Hearing loss can be detected by audiologic testing, but the logistics of obtaining formal audiograms are often challenging and obtaining serial evaluations for patients on prolonged treatments even more so. The Mimi Hearing App is a smartphone application that allows one to obtain self-administered audiograms. The purpose of this study was to evaluate the accuracy and precision of self-administered audiograms using this smartphone application.

**Figure:**
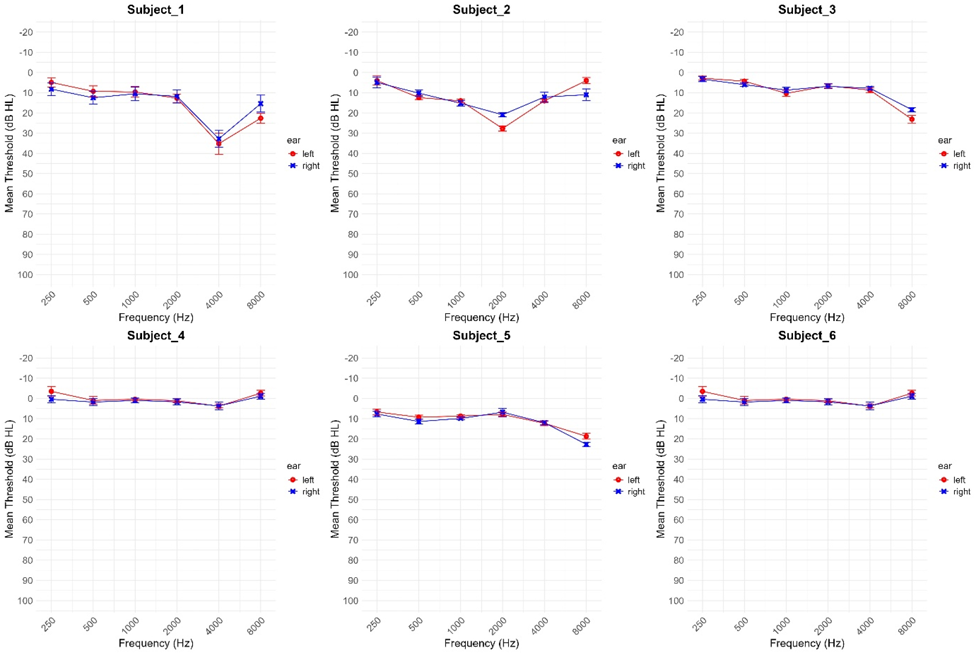

**Table 1.**
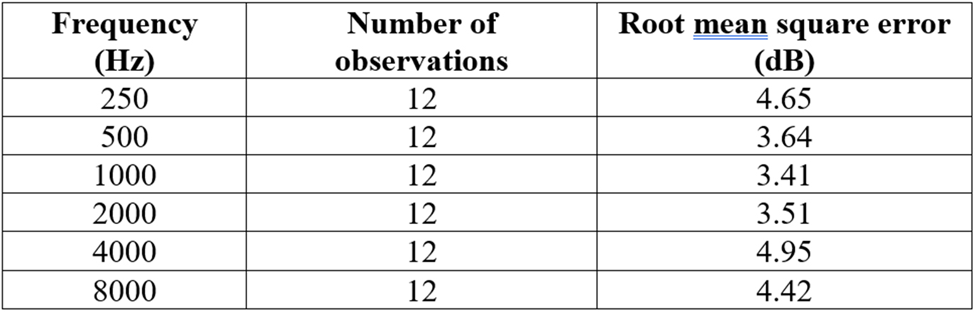
Precision of measurement of hearing loss by self-administered audiograms using the Mimi Hearing App on repeat testing

**Methods:**

Personnel working in the department of infectious diseases at Cleveland Clinic who had iPhones, and wired or wireless headphone devices compatible with iPhones, were invited to participate in the study. Each study subject was asked to record 10 self-administered audiograms on their iPhone, on 10 separate days, using the Mimi Hearing Application, and on completion of these, have a formal audiogram done by a Cleveland Clinic audiologist. Accuracy and precision of the self-administered audiograms at 250, 500, 1000, 2000, 4000, and 8000 Hz, were evaluated using the formal audiogram result as the gold standard.

**Table 2.**
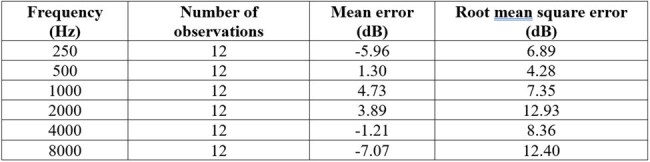
Accuracy of measurement of hearing loss by self-administered audiograms using the Mimi Hearing App compared to formal audiologic assessments

**Results:**

Sixty self-administered audiograms were available from 6 subjects. One subject was in the age range 30-39, two in the range 40-49, two in the range 50-59, and one in the range 60-69. Three were male and three were female. Summarized audiograms from the 10 readings for each subject, depicting mean readings with standard error bars at each tested frequency, are shown in Figure 1. The measurements from the self-administered audiograms were very precise, with a root mean square error of less than 5 dB at each tested frequency (Table 1). When compared to measurements on formal audiologic testing, the mean error ranged from -7.07 to 4.73 dB for the various tested frequencies (Table 2).

**Conclusion:**

Self-administered audiograms using the Mimi Hearing Application on a iPhone provide accurate measurements of hearing acuity with a high degree of precision between measurements.

**Disclosures:**

All Authors: No reported disclosures

